# First report on molecular identification of *Fusarium *species causing fruit rot of mandarin ( *Citrus reticulata*) in Bangladesh

**DOI:** 10.12688/f1000research.26464.2

**Published:** 2023-02-15

**Authors:** Mohammed Faruk Hasan, Mohammed Asadul Islam, Biswanath Sikdar

**Affiliations:** 1 Department of Genetic Engineering and Biotechnology, Faculty of Biological Sciences, University of Rajshahi, Rajshahi, 6205, Bangladesh; 2 Professor Joarder DNA & Chromosome Research Lab., Dept. of Genetic Engineering and Biotechnology, University of Rajshahi, Rajshahi, 6205, Bangladesh

**Keywords:** Citrus reticulata, Fruit Rot, Fusarium sp., PCR, ITS rRNA gene

## Abstract

**Background:** Fusarium rot is a newly introduced, devastating disease of citrus fruits. The current investigation was undertaken to characterize the microbes responsible for fruit rot in *Citrus reticulata*.

**Methods:** Pathogens were isolated from infected citrus fruits using morphological and molecular approaches. For confirmation of the isolated fungi, polymerase chain reaction (PCR) amplification and internal transcribed spacer gene sequencing techniques were used.

**Results:** The isolated fungus was grown on potato dextrose agar for three days and it produced clamydospores, hyphae and macroconidia. PCR amplification of isolated fungal DNA gave a 650 bp product. The sequence obtained from isolated fungi had 99.42% similarity with the reference *Fusarium concentricum* sequence in NCBI GenBank. The obtained sequence was deposited in GenBank (Accession No. MT856371). Two isolates showed virulence capability on fresh guava, sweet orange and tomato fruits, which confirmed species identification and Koch’s postulates. Artificially inoculated fungal species grown on tested fruits showed typical *Fusarium* species symptoms.

**Conclusions:** Outcomes of the present study are beneficial for the detection of this detrimental disease in postharvest *Citrus reticulata *fruits. Further research is needed for the control of this economically important disease. This is the first study of fruit rot in *Citrus reticulata *caused by *Fusarium* in Bangladesh.

## Introduction

*Citrus reticulata* Blanco, commonly known as mandarin, is an oblate fruit resembling other oranges, belonging to the family of Rutaceae ( Ahmed *et al*., 2014) and originating from hybridization with *Citrus maxima* ( Wang *et al*., 2018). Citrus fruits contain different vitamins, minerals and trace elements. *Citrus aurantifolia* fruits are usually eaten fresh or used in salads and also used as flavoring in some liqueurs ( Morton, 1987). In traditional medicine, they are also used for the treatment of rheumatoid arthritis and obesity ( Srinivasan *et al*., 2008).

*Fusarium* species can cause superficial infections in plants and animals with high mortality in persistently and severely neutropenic patients ( Dignani & Anaissie, 2004). *Fusarium* species are highly competent at contamination, possessing several mycotoxins ( O’Donnell *et al*., 2009) and different fruits decay in different storage and postharvest conditions ( Whiteside *et al*., 1988).

The novel *Fusarium* fungi were isolated and identified through applying advanced methods on different crops from different countries ( Aktaruzzaman *et al*., 2018; Al-Najada & Gherbawy, 2015; Geiser *et al*., 2004; Sun *et al*., 2018).

*Fusarium* species are one of the most imperative pathogenic fungi responsible for fruits rots of citrus causing lose their market value ( Ezrari *et al*., 2022 Numerous *Fusarium* species have been reported with citrus and other fruits decay in different countries ( Fogliata *et al*., 2013; Kurt *et al*., 2020; Mahmud *et al*., 2021; Paul *et al*., 2022). *Fusarium* fruit rot is a very common and destructive problem for mandarin due to harvesting and marketing in Bangladesh ( Ahmed *et al*., 2014).

To the best of our knowledge, there is no report of *Fusarium* based mandarin fruit rot in Rajshahi, Bangladesh. Therefore, this study aims to give a clear understanding of the *Fusarium* species association with mandarin rot in Bangladesh using molecular approaches.

## Methods

### Fungi isolation from the infected fruits

**Figure 1.**
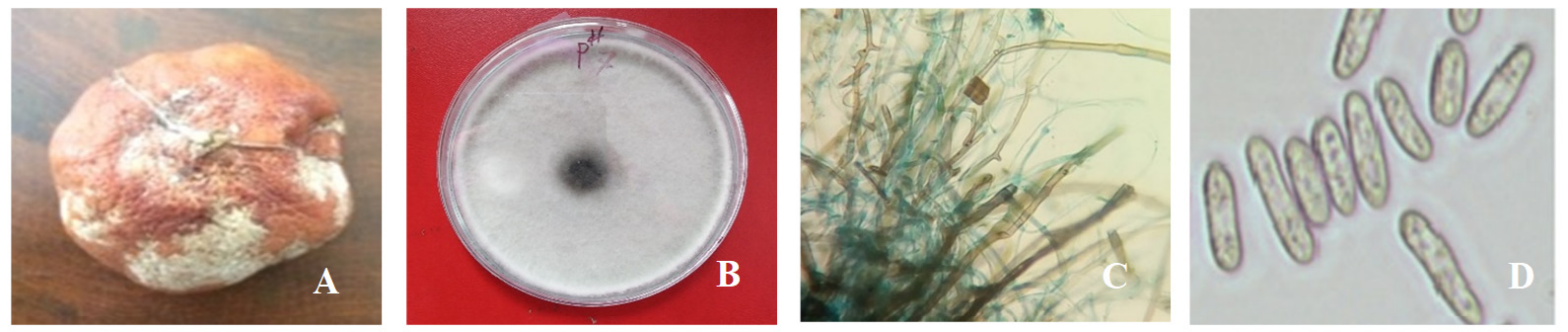
Naturally infected postharvest mandarin fruit showing symptoms of Fusarium rot and morphological phenotypes (F1). ( **A**) Infected mandarin fruit; ( **B**) fungus growth; ( **C**) clamydospores, hyphae, appressoria; and ( **D**) conidia under the microscope from seven-day-old culture at 35°C on potato dextrose agar.

***Research location and samples collection.*** The research was conducted at Professor Joarder DNA and Chromosome Research Lab., Department of Genetic Engineering and Biotechnology, University of Rajshahi, Bangladesh during 2018 to 2019. The ten different rotten *Citrus reticulata* fruits ( Figure 1A) were collected from fruit market in Rajshahi, Bangladesh. Out of 10 fruits, three showed symptoms of rot which were used for pathogen isolation.

***Fungi isolation from the infected fruits.*** Collected fruits were cleaned under running tap water to remove foreign agents and kept in a Biosafety Cabinet (Esco, Singapore). Moreover, the fruits were disinfested with 1% sodium hypochlorite (NaOCl) for 30 seconds, followed by five rinses in autoclave distilled water. Disinfested tissue was excised and plated on potato dextrose agar (PDA) (Hi-Media, India) at 35°C in the dark for three days. The colonies showing typical morphological characteristics including, colony color, pigmentation, growth rate and size of macroconidia of *Fusarium* species were selected ( Hafizi *et al*., 2013) and isolated using the single spore technique ( Chowdhury *et al*., 2019). Isolated colonies were transferred onto a Petri plate with PDA and incubated for seven days at 35°C in dark conditions. Isolates were grouped into two on the basis of morphological color (blackish color in the first group and whitish in the second group). Finally, one isolate from each of the two groups was selected for morphological and molecular analysis.

### Morphological characterization of isolates

The selected fungal colony was characterized by macromorphological and micromorphological investigation ( Al-Najada & Gherbawy, 2015). The isolate was sub-cultured in fresh PDA medium and three-day-old cultures were mounted using the lacto-phenol cotton blue (LPCB) staining method ( Sathya *et al*., 2017). The mounted microscope slide was covered with a cover slip and conidia were observed under a light microscope (LABOMED LX400, USA) at 40X magnification.

### Molecular characterization of *Fusarium* species

Genomic DNA was extracted from 15 gm of mycelia, collected from day three-day-old PDA cultures. DNA was extracted using a MaxMaxwell® 16 LEV Plant DNA Kit (Cat No. AS1420, Promega, USA) and DNA quantity and quality were checked using a NanoDrop 2000 Spectrophotometer (Thermo Scientific, USA).

To amplify the internal transcribed spacer (ITS) gene, primer pairs ITS4 (5 ^′^-TCCTCCGCTTATTGATATGC-3 ^′^) and ITS5 (5 ^′^-GGAAGTAAAAGTCGTAACAAGG-3 ^′^) were used ( Gardes & Bruns, 1993; White *et al*., 1990). The PCR reaction was performed using the method described by Hassan *et al*. (2018) using hot start green master mix (dNTPs, Buffer, MgCl2, Taq Pol) (Cat # M7432, Promega, USA). A total of 25 µl reaction volume containing 2 µl genomic DNA, 2.5 µl 1X PCR buffer, 1.0 µl MgCl _2_, 1.5 µl dNTPs, 0.5 µl of each primer, 0.5 µl of *Taq* polymerase and 16.5 µl of deionized water was used. The PCR was programmed with an initialization step at 95°C for 2 min, followed by 32 cycles of denaturation at 95°C for 30 seconds, primer annealing at 48°C for 30 seconds, and extension at 72°C for 45 seconds and a final extension at 72°C for 10 minutes using a G02 GeneAtlas PCR machine (Astec, Japan). PCR products were run by horizontal electrophoresis (Mini-Gel, CBS Scientific, USA) on 1% agarose (Cat # V3125, Promega, USA) gel with 0.5% ethidium bromide solution (Cat # H5041, Promega, USA) in 1x TAE buffer (Cat # V4251, Promega, USA) using a 1kb DNA ladder (Cat # G5711, Promega, USA) as marker and visualized under alpha imager UV trans-illumination (Mini, Protein Simple, USA). PCR products were purified from the agarose gel, using the Wizard SV Gel and PCR Clean-Up System (Cat # A9281, Promega, USA).

Purified PCR products were sequenced commercially by Sanger sequencing (Apical Scientific, Malaysia). Sequences were used in a search using the NCBI BLAST tool. Sequences were submitted to GenBank and compared with the GenBank database. A phylogenetic tree was constructed using MEGA 10 software using the neighbor joining method ( Kumar *et al*., 2016; Tamura *et al*., 2004).

### Virulence test

Virulence competency of the isolate was carried out using the method described by Tafinta *et al*. (2013). The surfaces of mature, fresh guava, lemon and tomato were sterilized using water and 70% ethanol. The fruits were holed using a 2 mm sized cork borer and selected fungal inoculums were aseptically placed in the holes. The inoculated samples and the control were placed in sterile polythene bags and incubated at 35°C for seven days in dark. Isolates from the fruits and colonies from the diseased lesions were sub-cultured in PDA. The isolated fungal stains were identified based on colony features, growth rates and pigmentation. For confirmation, genomic DNA was isolated from subcultured colonies, which were isolated from artificially infected fruits. PCR amplification was performed using same procedure described in our previous article ( Hasan *et al*., 2020) for virulence potency test through ITS rDNA gene amplification.

The study performed using a completely randomized design (CRD). Data were analyzed using Analysis of Variance (ANOVA) to see the differences between *Fusarium* species pathogenicity ( Kasiamdari & Sangadah, 2015). The analysis was performed using SAS software version 9.4.

## Results

### Morphological characterization

Collected samples were incubated on PDA medium following the single spore technique, and after seven days, white colored fungal colonies appeared ( Figure 1B).

Isolated fungus was whitish in color and produced clamydospores, hyphae, appressoria and macroconidia ( Figure 1C–D) at day three on PDA medium. Isolates produced microconidia that were 0-septate, oval, obovoid with a truncate base, elliptical or reniform. Macroconidia were sporodochia and fusiform. Chlamydospores were monophialides.

### Molecular characterization

**Figure 2.**
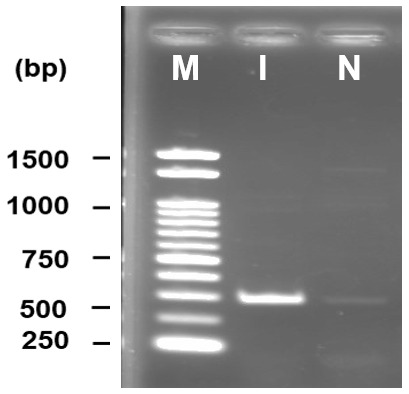
PCR amplification of internal transcribed spacer region yielded ~650 bp product (F1). 1kb DNA marker is used for size determination, M - marker, I - isolate, N - negative control.

DNA amplification through PCR produced a bright band at approximately 650 bp where a 1kb DNA ladder was used as marker. No band was found in the negative control where water was used instead of template DNA ( Figure 2).

**Figure 3.**
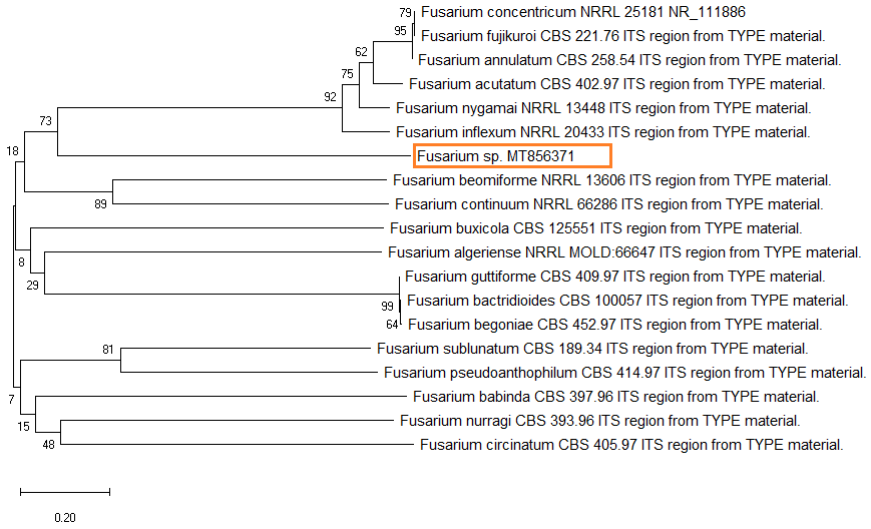
Phylogenetic tree based on the internal transcribed spacer region of rRNA showing closest relatives of fungal species isolated from citrus fruit samples (F1). The tree was constructed by neighbor joining method. The scale bar on the rooted tree indicates a 0.20 substitution per nucleotide position.

The dendrogram tree showed a close relationship with *Fusarium concentricum* and dissimilarity with *Fusarium begoniae* ( Figure 3). Therefore, molecular identification confirmed the isolates as *Fusarium* sp. The sequence of the total isolate was compared to *Fusarium* sequences in GenBank using BLASTN, which revealed closely related sequences and 99.42% homology with the reference sequence for *F. concentricum* (Accession No. NR_111886.1).

### Virulence test

**Figure 4.**
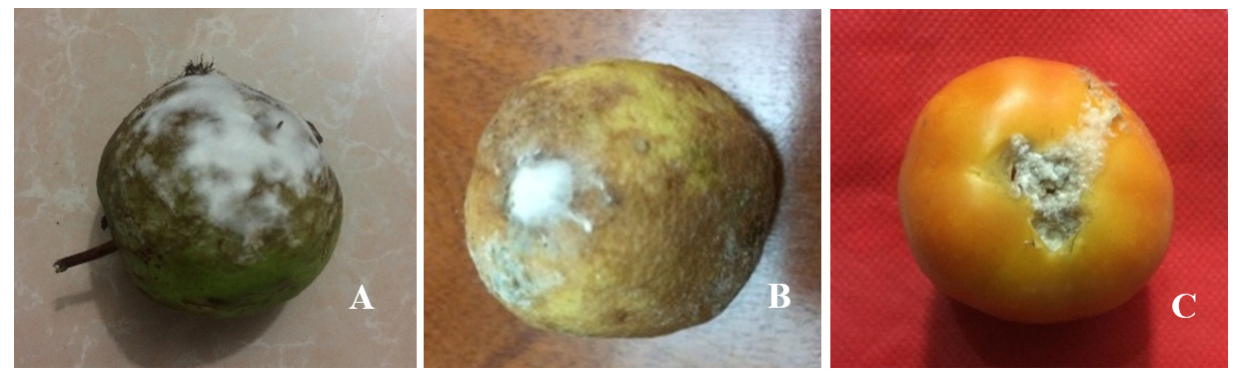
Fusarium rot symptoms in artificially inoculated fruits (F1). ( **A**) Guava, ( **B**) lemon and ( **C**) tomato, the image was taken 10 days after inoculation.

**Figure 5.**
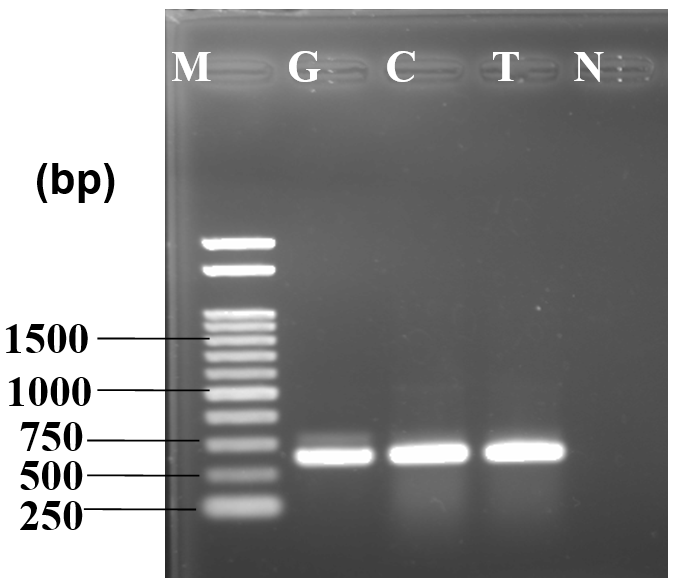
PCR amplification of internal transcribed spacer region yielded ~650 bp product for virulence test. 1kb DNA marker is used for size determination of inoculated fungus in different fruit samples. M - marker, G - guava, C - lemon, T - tomato, N - negative control.

The virulence test was conducted to characterize the fungus as pathogenic or saprophytic on mature, fresh and healthy guava, lemon and tomato. All fruits showed similar morphological characteristics of *Fusarium* symptoms ( Figure 4A–C). Isolated ribosomal DNA (rDNA) of fungus from artificially inoculated guava, lemon and tomato showed clear bands of approximately 650bp in length ( Figure 5).

## Discussion

Traditionally identification based on colony morphology, conidial morphology and other phenotypic characteristics has been used previously for different fungi of citrus fruits ( Leslie & Summerell, 2006; Tafinta *et al*., 2013). Further confirmation of the isolated fungi using advanced morphological and molecular approaches is required for characterization and differentiation of closely related *Fusarium* species ( Geiser *et al*., 2004). rDNA sequences of *Fusarium,* isolated from eggplant, lemon and onion (frequencies of occurrence ranging from 40% to 100%) were reported by Al-Najada & Gherbawy (2015).

The present *Fusarium* sp. responsible for mandarin fruit rot was identified using morpho- molecular approaches. *Fusarium* appeared as white or blackish-white and showed chlamydospores and macroconidia on PDA after seven days of culture. Huda-Shakirah *et al.* (2020) found 3–5 long, thin walled, septate macroconidia on a *F. concentricum* fugal stain under microscopic observation, which supports our present findings. Zhu *et al*. (2014) also found similar morphological characteristics for *Fusarium* isolated from dry root rot of citrus, lemon rot, dragon fruit rot, sugarcane wilt, eggplant rot and onion rot (frequencies of occurrence ranging from 40% to 100%) were reported by several researcher ( Al-Najada & Gherbawy, 2015; Ezrari *et al.,* 2022; Fogliata *et al.,* 2013; Paul *et al.,* 2022).

The present *Fusarium spp.* responsible for mandarin fruit rot was identified using morpho- molecular approaches. To confirm the morphological characterization, mandarin isolate of Fusarium were culture on PDA medium for seven days. *Fusarium* appeared as white or blackish-white and showed chlamydospores and macroconidia on PDA after seven days of culture. Microconidia were septate, oval and elliptical. Macroconidia were sporodochia and fusiform. Huda-Shakirah *et al.* (2020) found 3-5µm long, thin walled, septate macroconidia on a *F. concentricum* fugal stain under microscopic observation, which supports our present findings. Zhu *et al*. (2014) also found similar morphological characteristics for *Fusarium* isolated from *Eleocharis dulcis*. To confirm the individuality of the isolated fungi, the ITS4 and ITS5 were amplified using primers ITS4F/ITS5R. PCR amplification of ITS regions of the isolated fungal strains gave ~ 650 bp products in size and the sequences showed 99.42% similarity with the *Fusarium concentricum* sequence in the database. Kurt *et al*. (2020) obtained approximately 538 bp PCR amplicon on *Fusarium* isolated from mandarin using ITS regions. Huda-Shakirah *et al.* (2020) reported 99.53% similarity with *Fusarium concentricum* isolated from *Hibiscus sabdariffa*. These results are very similar to the present findings. Phylogenetic analysis was done using comparative analysis with different ITS regions of sequences published in NCBI database. Present findings of phylogenetic analysis showed that isolates of *Fusarium* species were in same clade with robust bootstrap support. Results of PCR products and ITS sequencing confirm the isolated fungus as *F. concentricum*, which is supported by some other researcher’s findings ( Chowdhury *et al*., 2019; Ezrari *et al*., 2022; Hasan *et al*., 2020; Hyun *et al*., 2000; Paul *et al*., 2022). *Fusarium* species are considered one of the most varied fungal species. It was related with numerous plant hosts and is a thoughtful risk to *Citrus reticulata* production due to rot. It is also responsible for twig rot, decline dieback, blight and wilt of citrus. *Fusarium* rot of citrus is fetching a significant worldwide problem. This report for the first time confirmed that *Fusarium* species are the causative microbe of citrus fruit rot in Bangladesh. This report has significance to develop suitable management practices to control the *Fusarium* rot diseases of citrus fruits.

## Conclusions

Fusarium fruit rot is a big problem for the citrus fruit industry in Bangladesh. In this study, *Fusarium* species were found to cause mandarin fruit rot. Moreover, pathogenicity was confirmed according to Koch’s postulates using three different types of fresh fruits. *Fusarium* species fruit rot leads to declines in the Bangladeshi fruit industry as well as fruit markets. Therefore, the current study may help the development of control measures for postharvest mandarin rot.

## Data Availability

*Fusarium* sp. pure cultured isolate containing small subunit ribosomal RNA, internal transcribed spacer 1 and 5.8S ribosomal RNA on GenBank. Accession number, MT856371: https://www.ncbi.nlm.nih.gov/nuccore/MT856371.1?report=genbank. Figshare: PCR amplification of ITS region yielded ~650 bp product. https://doi.org/10.6084/m9.figshare.13014209.v1 ( Hasan, 2020a). This project contains the following underlying data: Figure 2.jpg (original, unedited gel image from Figure 2) Figshare: PCR amplification of ITS region for virulence test. https://doi.org/10.6084/m9.figshare.13008458.v1 ( Hasan, 2020b). This project contains the following underlying data: Gel doc.2.jpg (original, unedited gel image from Figure 5) Figshare: Molecular identification of Fusarium species causing fruit rot. https://doi.org/10.6084/m9.figshare.12990746.v1 ( Hasan, 2020c). This project contains the following underlying data: Micro.imag.1.jpg (original, unedited microscopy image showing clamydospores, hyphae and appressoria from Figure 1C) Micro.imag.2.jpg (original, unedited microscopy image showing conidia from Figure 1D) Data are available under the terms of the Creative Commons Attribution 4.0 International license (CC-BY 4.0).
