# Supplementary material for: First report on molecular identification of Fusarium species causing fruit rot of mandarin ( Citrus reticulata) in Bangladesh
Source: F1000Res. 2023 Feb 15;9:1212. Originally published 2020 Oct 8. [Version 2] doi: 10.12688/f1000research.26464.2 (PMC9971657; doi:10.12688/f1000research.26464.2)
Supplement: Peer review history

# Peer review: Reviewer response for version 2

**DOI:** 10.5256/f1000research.142811.r163583

**Published:** 2023-02-27

**Authors:** Barbara Sawicka

**Affiliations:** 1 Department of Plant Production Technology and Commodities Science, University of Life Science in Lublin, Lublin, Poland

The work has been corrected and in its current version it is suitable for printing.

Is the work clearly and accurately presented and does it cite the current literature?

Partly

If applicable, is the statistical analysis and its interpretation appropriate?

No

Are all the source data underlying the results available to ensure full reproducibility?

Partly

Is the study design appropriate and is the work technically sound?

Partly

Are the conclusions drawn adequately supported by the results?

Partly

Are sufficient details of methods and analysis provided to allow replication by others?

No

Reviewer Expertise:

NA

I confirm that I have read this submission and believe that I have an appropriate level of expertise to confirm that it is of an acceptable scientific standard.

# Peer review: Reviewer response for version 1

**DOI:** 10.5256/f1000research.29219.r158099

**Published:** 2022-12-19

**Authors:** Barbara Sawicka

**Affiliations:** 1 Department of Plant Production Technology and Commodities Science, University of Life Science in Lublin, Lublin, Poland

The work entitled: "The first report on the molecular identification of Fusarium species causing mandarin fruit rot (Citrus reticulata) in Bangladesh" is an important contribution to the research on identifying the causes of mandarin fruit rot. However, the authors made several mistakes that need to be corrected before the work is published.

 Detailed notes:

 1) The abstract is properly structured.

 2) The introduction should introduce the reader to the subject of the work and end with a clear purpose of the work.

 3) The purpose of the research should be clearly explained. The authors should also put forward an alternative research hypothesis to the null hypothesis in order to verify it later in the work.

 4) The methodology of work should be extended to the methodology of sampling for testing (the number of samples and isolates should be sufficient to make statistical calculations) and the methodology of statistical calculations.

 5) Research results should be thoroughly discussed with statistical justification.

 6) The "Discussion" section needs to be expanded. It should include references to the latest literature in the field.

 7) The application should be general and summative and should include a clear indication for the manufacturing practice. In addition, it should contain an indication (proposal) for the future.

 8) The list of literature should be enriched with the latest items in the researched area.

Is the work clearly and accurately presented and does it cite the current literature?

Partly

If applicable, is the statistical analysis and its interpretation appropriate?

No

Are all the source data underlying the results available to ensure full reproducibility?

Partly

Is the study design appropriate and is the work technically sound?

Partly

Are the conclusions drawn adequately supported by the results?

Partly

Are sufficient details of methods and analysis provided to allow replication by others?

No

Reviewer Expertise:

agriculture, agronomy, commodity sciences, food safety, plant protection

I confirm that I have read this submission and believe that I have an appropriate level of expertise to confirm that it is of an acceptable scientific standard, however I have significant reservations, as outlined above.

# Response

**Published:** 2023-01-02

**Authors:** Md. Faruk Hasan

**Affiliations:** University of Rajshahi, Bangladesh

Dear Reviewer,

 Thank you for your valuable comments and suggestions about our manuscript. We make some correction according to your comments in different parts of our manuscript which are marked as red color. We are submitting the new version of our MS.

 Regards

 Authors

# Peer review: Reviewer response for version 1

**DOI:** 10.5256/f1000research.29219.r73841

**Published:** 2021-01-07

**Authors:** Shuvra Kanti Dey

**Affiliations:** 1 Department of Microbiology, Jahangirnagar University, Dhaka, Bangladesh

**General comment:**

 I found the article well-written and cohesive. I was happy to read about the whole article and I am glad to recommend for indexing with minor corrections.

**Specific comments:**

**Title and abstract: **Title is very informative and abstract gives good insight into the study.

**Introduction:** Author describe the introduction very well. Importance of the study, background and objectives are very clear. It provided important information.

 Need to add some recent references.

**Methods:** I think this section was clearly describe with relevant references.

**Results:** This study describes the isolation and identification of a proposed new species of *Fusarium*. The isolate has been characterized using molecular approaches. The obtained sequence was deposited in GenBank ( **Accession No. MT856371**). PCR and sequencing confirm the isolated stain. At the genomic level, the proposed new species was **99.42%** similarity with the reference *Fusarium concentricum* sequence. Virulence test showed the fungus as pathogenic on healthy guava, lemon and tomato. Therefore, the proposed species meets the molecular and morphological requirements of being designated as a new species.

 Need to add outgroup in the phylogenetic tree.

Is the work clearly and accurately presented and does it cite the current literature?

Yes

If applicable, is the statistical analysis and its interpretation appropriate?

I cannot comment. A qualified statistician is required.

Are all the source data underlying the results available to ensure full reproducibility?

Yes

Is the study design appropriate and is the work technically sound?

Yes

Are the conclusions drawn adequately supported by the results?

Yes

Are sufficient details of methods and analysis provided to allow replication by others?

Yes

Reviewer Expertise:

I confirm that I have read this submission and believe that I have an appropriate level of expertise to confirm that it is of an acceptable scientific standard.

I confirm that I have read this submission and believe that I have an appropriate level of expertise to confirm that it is of an acceptable scientific standard.
